# Microperimetric Assessment after Epiretinal Membrane Surgery: 4-Year Follow-Up

**DOI:** 10.1155/2016/7030791

**Published:** 2016-03-21

**Authors:** Marco Dal Vecchio, Carlo Lavia, Marco Nassisi, Federico M. Grignolo, Antonio M. Fea

**Affiliations:** Department of Surgical Sciences, Eye Clinic, University of Turin, 10122 Turin, Italy

## Abstract

*Purpose*. To investigate retinal function using microperimetry in patients affected by idiopathic epiretinal membrane (iERM) and cataract who underwent combined surgery: 4-year follow-up.* Design*. Prospective, interventional case series.* Methods*. 30 eyes of 30 consecutive patients with iERM and age-related cataract underwent 25-gauge vitrectomy and cataract surgery. At baseline, 90 and 180 days, and 1 and 4 years, we examined retinal mean sensitivity (MS), retinal mean defect (MD), fixation stability, and frequency of microscotomas using MP1 microperimetry. Best-corrected visual acuity (BCVA) and central retinal thickness (CRT) using a spectral domain optical coherence tomography (SD-OCT) were also performed.* Results*. All patients completed 1-year follow-up, while 23 patients reached last follow-up. Baseline MS and MD (10.48 ± 4.17 and −9.18 ± 4.40 dB) significantly changed at one year (12.33 ± 3.66 and −7.49 ± 3.31 dB, *p* < 0.01), at four years (14.18 ± 3.46 and −4.66 ± 2.85, *p* < 0.01), and between one and four years (*p* < 0.01) after surgery. Compared to baseline, CRT and BCVA significantly changed at one year and remained stable at four years. No variations were observed in fixation stability and frequency of microscotomas compared to baseline.* Conclusions*. Long-term follow-up using microperimetry seems useful to evaluate patients after iERM surgery: retinal sensitivity changes even when BCVA and CRT remain stable.

## 1. Introduction

Idiopathic macular epiretinal membrane (iERM) is a relatively common disorder, with an incidence reaching 12% in those older than 70 years [1].

Different hypothesis has been advanced about iERM pathogenesis. On one hand, iERM seems to begin with microfractures in the inner retina after posterior vitreous detachment, while, on the other hand, it seems to occur when the external layer of the posterior vitreous cortex remains attached to the macula. iERM can vary from a single layer to a thick, multilayer fibrocellular proliferation shrinking the retinal surface [2].

Although patients with iERM can be asymptomatic at the beginning, they may complain of various degree of visual symptoms: distortion of lines (metamorphopsia), decreased visual acuity, macropsia, micropsia, and monocular diplopia.

In symptomatic patients, the surgical removal of the epiretinal membranes with pars plana vitrectomy (PPV) is the gold standard surgical procedure [3, 4].

Peeling of the ILM during surgery is still debated. According to Bu et al., it could be advisable to remove ILM to reduce ERM recurrence by eliminating the scaffold for the proliferation of fibrocellular tissue [2]. On the contrary, Liu et al. reported that ILM + ERM peeling compared to ERM peeling alone, achieved better best-corrected visual acuity (BCVA) 12 months after surgery but not after 18 months, showing that a longer follow-up would be advisable [5].

Quality of life, contrast, and color sensitivity are becoming useful parameters in the workup of patients affected by iERM, together with visual acuity and retinal morphology.

Microperimetry provides information on foveal fixation, macular sensitivity, and depth of central macular defects and is gaining interest in the assessment of various retinal diseases [6–12].

Microperimetry, before and after iERM surgery, can help the surgeon in evaluating which kind of patients could benefit from surgery and what could be their prognosis. Moreover, thanks to the autotracking system that corrects involuntary eye movements and the possibility to do follow-up examinations, it allows a greater reliability and reproducibility of the tests than automated perimetry [13, 14].

Long-term follow-up is a useful tool to evaluate the effectiveness of a treatment, especially when a total consensus about the timing of a surgical procedure is still not available.

Aim of the study was to investigate the potential recovery of retinal functions assessing the microperimetric outcomes in patients affected by iERM and cataract who underwent 25-gauge PPV with ILM peeling combined with phacoemulsification and intraocular lens (IOL) implantation in a long-term follow-up. CRT and visual acuity outcomes were also analyzed.

## 2. Materials and Methods

This interventional open label study has been approved by the Local Institutional Ethics Committee and Review Board and registered on ClinicalTrials.gov (number NCT01771939). The research adhered to the tenets of the Declaration of Helsinki and written informed consent was obtained from all patients before participation in the study.

From October 2009 to July 2010, 30 eyes of 30 consecutive patients affected by idiopathic epiretinal membranes and various degree of cataract were recruited for the study. All patients underwent a combined 25-gauge PPV, phacoemulsification, and IOL implantation.

Inclusion criteria were iERM clinical finding, macular thickness > 250 *μ*m as measured by Spectral-Domain OCT (SD-OCT, RTVue 100, Optovue, Fremont, CA), presence of metamorphopsia at the Amsler grid chart, and a visual acuity loss of 2 Snellen lines in the last six months.

Exclusion criteria were glaucoma, corneal or lens opacities that precluded an acceptable retinal visualization, ocular axial length > 25 mm (measured with A-Scan biometry), epiretinal membrane secondary to trauma or vascular diseases, any macular degeneration, diabetic retinopathy, and/or previous ophthalmic surgery.

All patients underwent a routine ophthalmic examination (before and at 1, 7, 30, 90, 180, and 360 days after surgery) including slit-lamp biomicroscopy, BCVA with the Early Treatment Diabetic Retinopathy Study (ETDRS) score at 4 meters, dilated fundus examination, and intraocular pressure measurement using Goldmann Tonometry.

OCT examination calculating central retinal thickness (CRT) in the central mm using the Macular Map 5 × 5 mm (MM5) and microperimetry with the MP1 (Nidek Technologies, Padova, Italy) were performed before surgery and at 90, 180, and 360 days after surgery.

Patients were called back to our center four years after the intervention to assess long-term functional and morphological outcomes.

### 2.1. Surgical Procedure

An expert vitreoretinal surgeon (MDV) performed all surgical procedures. After the insertion of three 25-gauge cannulas, through a 2.75 mm clear-cornea incision, phacoemulsification of the lens was performed. A foldable intraocular lens (IOL) Akreos AO (Bausch and Lomb, Rochester, NY, USA) was implanted in all cases.

The core vitrectomy was performed with the Accurus machine 25 G+ (Alcon Laboratories, Fort Worth, TX, USA). The posterior hyaloid membrane, if not spontaneously detached, was dyed with micronized triamcinolone acetonide (IVT®, BIOOS, Italy), separated by aspiration, and removed. After the extension of vitrectomy to the ora serrata the MembraneBlue-Dual dye (DORC, Zuidland, Netherlands) was used in order to peel the ERM and the ILM within the vascular arcades, with a 25-gauge micro forceps (Alcon Laboratories). A second stain with MembraneBlue-Dual dye was applied to check whether the ILM peeling was completed. An accurate inspection of the peripheral retina and the photocoagulation of eventual retinal holes, tears, or rhegmatogenous degenerations was followed by the exchange of the balanced salt solution (BSS) with filtered air and cannulas removal.

Tobramycin 0.3% and dexamethasone 0.1% eye drops were prescribed 3 times a day for a month after surgery.

### 2.2. Microperimetry

All images were acquired by an expert examiner.

Patient sat in front of the MP1 with the head carefully aligned in the chin rest and against the forehead strap.

All subjects underwent the exam under dim light conditions with dilated pupil in the study eye. Fellow eye was patched. After the baseline visit, all other examinations were performed using the “follow-up” mode.

A 10° grid of 40 spots was centered on the fovea region. Stimulus size was Goldmann III white spot, with a stimulus duration of 200 ms. Threshold strategy was HFA 4-2, and background luminance was 1.27 cd/m^2^ (4 asb).

Stimulus attenuation ranged from 0 dB that represents the instrument's maximum stimulus luminance to 20 dB that represents the minimum stimulus luminance.

The fixation point was set as single white cross of 1° of spatial width. The automatic eye tracker was used to compensate eye movements and to calculate horizontal and vertical shifts relative to a reference frame during the examination. The recorded fixation points were classified into three categories for fixation stability analysis (stable, relatively unstable, and unstable). Fixation was regarded as “stable” if more than 75% of the fixation points were inside the 2° diameter circle, as “relatively unstable” if <75% were inside the 2° diameter circle but more than 75% inside the 4° diameter circle, and as “unstable” if <75% were inside the 4° diameter circle. Fixation stability was considered for statistical analysis.

Mean sensitivity (MS), mean defect (MD), and the total number of absolute scotoma locations (points with a threshold value of 0 dB) in the 10° central area were evaluated.

Mean retinal sensitivity is the arithmetic mean of all the measured absolute thresholds expressed in dB; mean retinal defect is the arithmetic mean of all local defects, including the values above the upper limits (expressed in dB).

### 2.3. Outcome Measures

Primary outcome measures were the changes in MS and MD.

Secondary outcomes were the changes in BCVA, CRT, fixation stability at 2° and 4°, and frequency of microscotomas in the 10° area.

### 2.4. Statistical Analysis

Statistical analysis was carried out with Analyse-it statistical software for Microsoft Excel (version 2.26; Analyse-it Software, Leeds, UK). The Shapiro-Wilk test was employed to verify if data were normally distributed. Differences in BCVA, CRT, and microperimetry values before and after surgery were determined with the Wilcoxon signed-ranks tests.

The values of *p* < 0.05 were considered statistically significant.

Data are presented as mean ± standard deviation.

## 3. Results

30 patients affected by iERM and age-related cataract were enrolled. There were 18 males and mean age was 70.8 ± 8 years. All patients completed the 1-year follow-up visit, whereas 23 of them (76.6%) completed the 4-year follow-up.

Preoperative demographics are reported in Table 1.

7 patients were unable to attend the last follow-up visit: 3 had moved to another city, 2 were unable to come to our center, and 2 of them died.

In the 23 patients who completed the 4-year follow-up, no significant differences at all study visits were detected compared to the 30 patients who completed the one-year visit.

All data were normally distributed.

**Figure 1:**
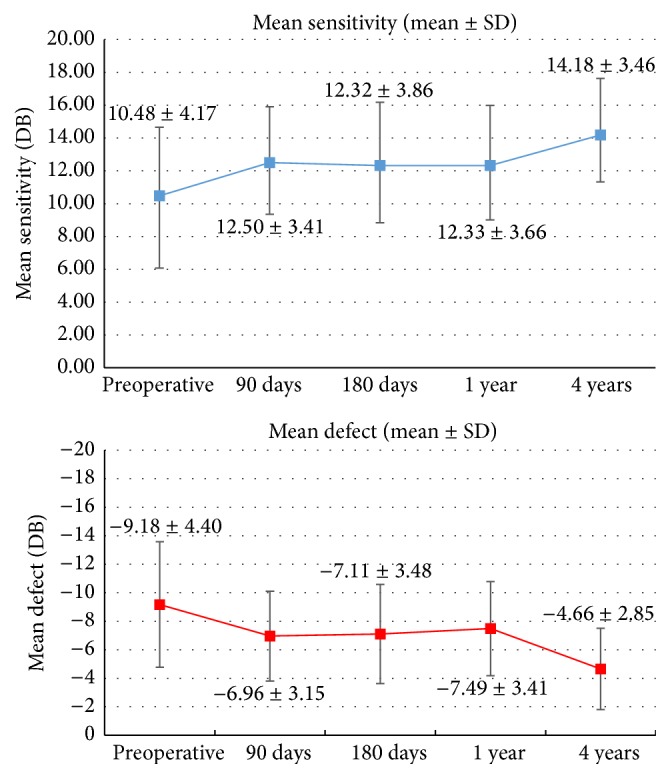
Mean sensitivity and mean defect values before and after surgery (mean ± standard deviation).

Microperimetric MS and MD trends are reported in Figure 1. Secondary outcomes trends are reported in Table 2.

Statistical analysis is resumed in Table 3.

### 3.1. Intraoperative and Postoperative Complications

No significant intraoperative complications were recorded. In one case (3.3%), a laser photocoagulation was required due to a little iatrogenic hole beyond the vascular arcades, without consequences for the macular function or the visual acuity.

19 patients (63%) developed posterior lens capsule opacity and underwent YAG laser capsulotomy before follow-up exams.

### 3.2. Best-Corrected Visual Acuity

Preoperative mean visual acuity significantly improved (*p* < 0.01) after surgery at both 1 and 4 years. The highest gain in mean visual acuity was documented at 4 years although no significant differences were found between 1 year and 4 years (*p* = 0.08).

Compared to baseline, at one and 4 years, 16/30 (53.3%) and 15/23 patients (65.2%) presented a gain in visual acuity > 2 ETDRS lines, respectively.

Mean BCVA change was of 3.1 ETDRS lines at one year and 3.3 ETDRS lines at 4 years.

### 3.3. OCT-CRT

Preoperative mean CRT significantly changed (*p* < 0.01) after surgery at both 1 and 4 years. Baseline mean value was 381.22 ± 69.86 *µ*m, while at one and four years 293.71 ± 47.66 *µ*m and 280.74 ± 39.57 *µ*m were, respectively, measured. In one patient, the CRT increased (31 *µ*m at one year and 18 *µ*m at 4 years) probably due to preoperative borderline CRT value (252 *µ*m). No significant changes were observed in mean CRT values between 1 and 4 years (*p* = 0.08).

### 3.4. Microperimetry

Significant differences (*p* < 0.01) were found between preoperative and postoperative data at one and four years for both mean retinal sensitivity and mean defect (Figure 1).

At baseline, one year, and four years the mean retinal sensitivity in the 10° central area was 10.48 ± 4.17 dB, 12.33 ± 3.66 dB, and 14.18 ± 3.46 dB, respectively.

At baseline, one year, and four years the mean retinal defect in the 10° central area was −9.18 ± 4.40 dB, −7.49 ± 3.31 dB, and −4.66 ± 2.85 dB, respectively.

Significant differences were found in MS and MD between one and four years after surgery (*p* < 0.001).

No significant differences in fixation stability in the central 2° and 4° were found at all visits. The same observation was made for the number of microscotomas in the central 10°.

## 4. Discussion

Surgical approach in symptomatic patients with iERM showed good results in terms of visual acuity and retinal function recovery with minimal surgical complications and no recurrence of pathology.

The application of dyes during vitreoretinal surgery improved visualization of ERM and the vitreoretinal interface. The toxic effect of dyes used during the peeling cannot be excluded. However, Trypan blue and Brilliant blue G used in this study are recognized safe staining agents with no or minimal toxic effects on retina at the concentrations used [15].

When patients present a certain degree of cataract, the association of PPV with cataract surgery enables a better visualization of the retina.

Moreover, PPV in phakic eyes determines a progression of nuclear sclerosis [16], and, after vitreoretinal surgery, phacoemulsification is more difficult and with a greater rate of complications [16, 17].

Our patients showed a significant and early improvement of functional outcomes and a reduction of CRT at OCT, as reported in the literature [18–20].

Although after iERM surgery BCVA continues to improve up to 2 years, best values are reached at about 1 year, in accordance with our findings, as no changes have been detected between 1 and 4 years [21–23].

As observed by Ripandelli et al. in patients who underwent ILM peeling, in our study the number of microscotomas in the 10° analyzed area improved after surgery, although not significantly [4].

With a steady fixation stability over time, MS and MD interestingly increased between 1 and 4 years, demonstrating a significant dissociation between BCVA and retinal sensitivity, as has already been demonstrated in other retinal pathologies, underlying the microperimetry capability to detect even subtle changes in patients' quality of life [6].

As in our study combined surgery was performed in all cases, it was impossible to determine if the immediate postoperative outcome improvements were completely associated with the iERM removal or with cataract extraction or both.

On the other hand, the double procedure removed an important potential confounding factor in the evaluation of the outcomes after surgery.

This study has some limitations: the small sample size analyzed might have influenced the power of the study, the incomplete follow-up of some patients (only 67% at four years), and the absence of a quantification of metamorphopsia (e.g., M-Chart) providing another quality of life parameter [24].

## 5. Conclusions

In conclusion, microperimetry proved an effective diagnostic tool in evaluating subtle changes in retinal function, undetectable with a visual acuity exam. Long-term follow-up after vitreoretinal surgery would be advised to reveal changes in retinal sensitivity affecting patients quality of life.

The use of high definition imaging techniques to analyze further aspects of this pathology such as correlation between long-term anatomical changes and microperimetric data are recommended.

## Figures and Tables

**Table 1:** Demographic characteristics.

Patients *n*/eyes *n*	30/30

Male/female	18 (60%)/12 (40%)

Age at enrollment, years (mean values ± standard deviation)	70.8 ± 8.0 (range 57–84)

Patients at 1 year follow-up	30 (100%)

Patients at 4 years follow-up	23 (76.7%)

**Table 2:** Secondary outcomes (mean values ± standard deviation; LogMAR: logarithm of minimal angle of resolution; CRT: central retinal thickness).

	Baseline	Day 90	Day 180	Day 360	4 years
LogMAR	0.65 ± 0.27	0.39 ± 0.22	0.38 ± 0.23	0.35 ± 0.20	0.29 ± 0.28
CRT (*µ*m)	376.39 ± 72.27	311.89 ± 65.87	298.17 ± 63.25	289.91 ± 54.61	278.57 ± 42.54
Fixation in central 4° (%)	80 ± 21.95	81.4 ± 20.79	87.33 ± 12.82	77.46 ± 23.52	76.26 ± 22.28
Fixation in central 2° (%)	94.46 ± 7.63	94.63 ± 7.67	97.63 ± 3.14	94.4 ± 9.96	92.08 ± 11.34
Microscotomas in central 10° (*n*)	2.6 ± 4.33	1.83 ± 3.66	1.86 ± 3.32	2.56 ± 4	3.65 ± 5.29

**Table 3:** Statistical analysis (MS: mean sensitivity; MD: mean defect; Fix: fixation; BCVA: best-corrected visual acuity; CRT: central retinal thickness).

*p* value–Wilcoxon test	MS	MD	10° microscotoma	Fix 2°	Fix 4°	BCVA	CRT
Preoperative versus 90 days	0.010	0.006	0.459	0.841	0.986	<0.001	<0.001
Preoperative versus 180 days	0.036	0.016	0.429	0.124	0.124	<0.001	<0.001
Preoperative versus 1 year	0.037	0.044	0.246	0.401	0.841	<0.001	<0.001
Preoperative versus 4 years	0.001	<0.001	0.267	0.070	0.174	<0.001	<0.001
1 year versus 4 years	0.001	<0.001	0.872	0.368	0.215	0.080	0.078
